# Constitution and By-Laws of the Cincinnati Dental Association

**Published:** 1860-02

**Authors:** 


### CONSTITUTION AND BY-LAWS OF THE CINCINNATI DENTAL ASSOCIATION.

### CONSTITUTION.

### ARTICLE I.

This Society shall be known by the name of the "Cincinnati Dental Association."

### ARTICLE II.

The objects of this Society shall be the mutual improvement and professional intercourse of its members.

### ARTICLE III.

SECTION 1. The officers shall consist of a President, Vice President, Recording and Corresponding Secretary, and Treasurer.

SEC. 2. The officers shall be elected semi-annually in September and March and shall serve until their successors are elected: the election shall be by ballot.

SEC. 3. It shall be the duty of the President to preside at all the meetings: and, with the concurrence of the Vice President or Secretary, to call extra sessions whenever occasion requires.

SEC. 4. It shall be the duty of the Vice President to preside in the absence of the President and to perform the duties devolving on that officer; and also to counsel with the President with difficult points.

SEC. 5. The Recording Secretary shall keep a record, (in a book provided for the purpose,) of the proceedings of all meetings; take charge of all papers and documents belonging to the Society and file the same; and serve notices.

SEC. 6. It shall be the duty of the Corresponding Secretary to conduct the correspondence of the Society not connected with its members; to keep a correct copy of all letters written by himself officially, and a file of all he may receive. He shall also report to the Society as often as any thing shall occur to him, officially, of interest to the Association.

SEC. 7. The Treasurer shall hold in trust for the Society any and all funds belonging to the same, and pay them out on the order of the Secretary, and collect all assessments and dues.

ARTICLE IV.

SEC. 1. The meetings shall be held at such time and place as a majority of those present may determine.

SEC. 2. Business details shall be governed by such by-laws as the Society may be pleased to adopt.

SEC. 3. Five active members shall constitute a quorum for the transaction of business.

ARTICLE V.

SEC. 1. Any reputable dental practitioner in the city of Cincinnati or vicinity may become an active member of this Association by receiving a vote of two-thirds of all the active members, signing the Constitution, and paying into the treasury the sum of two dollars.

SEC. 2. Any reputable dental practitioner, not a resident of Cincinnati or its vicinity, may become an honorary member of this Society by receiving a vote of three-fourths of the active members.

SEC. 3. Any reputable practitioner of medicine may become an honorary member of this Society by receiving a vote of two-thirds of the active members.

ARTICLE VI.

This Constitution may be altered or amended—notice of the same having been given one month previously—by a vote of two-thirds of the members of the Association.

### BY-LAWS.

ARTICLE I.

Honorary members shall be entitled to a seat at all meetings of the Society, and shall have the privilege of participating in all debates not involving pecuniary expenditures.

ARTICLE II.

The following shall be observed as the general order of proceedings of each meeting:

Reading minutes of previous meeting.

Election of new members.

Report of Committees.

Reading Essay and remarks on the same.

Discussion of the stated subject.

Written or verbal reports of cases.

Modes of practice,—Surgical, Medical, Operative and Mechanical.

Miscellaneous business.

Announcement by the President of Essayist and subject for discussion at the next ensuing meeting.

Adjournment.

ARTICLE III.

SEC. 1. The stated subject for discussion shall be designated by the Society, and the President may appoint one or more persons to lead in the debate or to prepare a paper on the same.

SEC. 2. No member shall be permitted to speak longer than ten minutes, or more than twice upon any one question without express permission of the Society.

SEC. 3. Any member may be called to order for personal recrimination, or for digressing from the subject of debate.

ARTICLE IV

These By-Laws may be altered or amended at any regular meeting of the Society by a vote of two-thirds of the members present: provided notice of such alteration or amendment shall have been given at a previous meeting.

